# *k_trans _*as a quantitative indicator of calf muscle perfusion at peak exercise

**DOI:** 10.1186/1532-429X-16-S1-P27

**Published:** 2014-01-16

**Authors:** David Lopez, Patrick Antkowiak, Craig H Meyer, Frederick H Epstein, Christopher M Kramer

**Affiliations:** 1 Medicine, University of Virginia, Charlottesville, Virginia, USA; 2 Biomedical Engineering, University of Virginia, Charlottesville, Virginia, USA; 3 Radiology and Medical Imaging, University of Virginia, Charlottesville, Virginia, USA

## Background

Quantitative contrast enhanced MRI (CE-MRI) may be valuable for calf perfusion assessment in peripheral arterial disease (PAD). The Kety equation describes the dynamic relationship between the concentration of gadolinium (Gd) in tissue and in arterial blood.*k_trans _*is the rate at which Gd enters the interstitial space and it is determined by blood flow (*F*) as well as the Gd extraction rate (*E*). We aimed to measure *k_trans _*at peak exercise in healthy (NL) and PAD subjects.

## Methods

Seven NL (age 53 ± 11, ankle brachial index (ABI) 1.0 ± 0.1, exercise time (ET) 353 ± 241 min) and 2 PAD subjects (age 80 ± 2, ABI 0.65 ± 0.05, ET 287 ± 67 min) were studied. A saturation recovery dual-contrast spoiled gradient echo (GRE) sequence was utilized to acquire the arterial input function (AIF) and muscle tissue function (TF). The pulse sequence was tested against Gd phantoms. AIF inversion time (TI) = 10 msec. TF TI 300 msec. Other parameters included delay between excitation pulses, 3.5 msec, matrix = 128 × 128, FOV = 180 × 180 mm, 8 mm thick, FA = 15°, TR = 768 msec, TE = 1.43 msec. A proton density image was acquired for signal normalization, FA = 5°. Subjects exercised using an MRI-safe plantar flexion ergometer until exhaustion in a Siemens Trio 3T scanner. At peak exercise 75 non-ECG-gated GRE measurements were obtained while infusing 0.1 mmol/kg of Gd-DTPA at 4 cc/s. Regions of interest were drawn on the muscle of interest and its feeding artery on the TF and AIF slices, respectively. Bloch equation modeling was performed to convert signal intensity into Gd-DTPA concentration. The AIF was fit to a gamma-variate function with an exponential washout term. Constrained Kety model deconvolution of the AIF from the TF was performed to calculate *k_trans _*using a Levenberg-Marquardt nonlinear least squares optimization implemented in Matlab.

## Results

**Figure 1:**
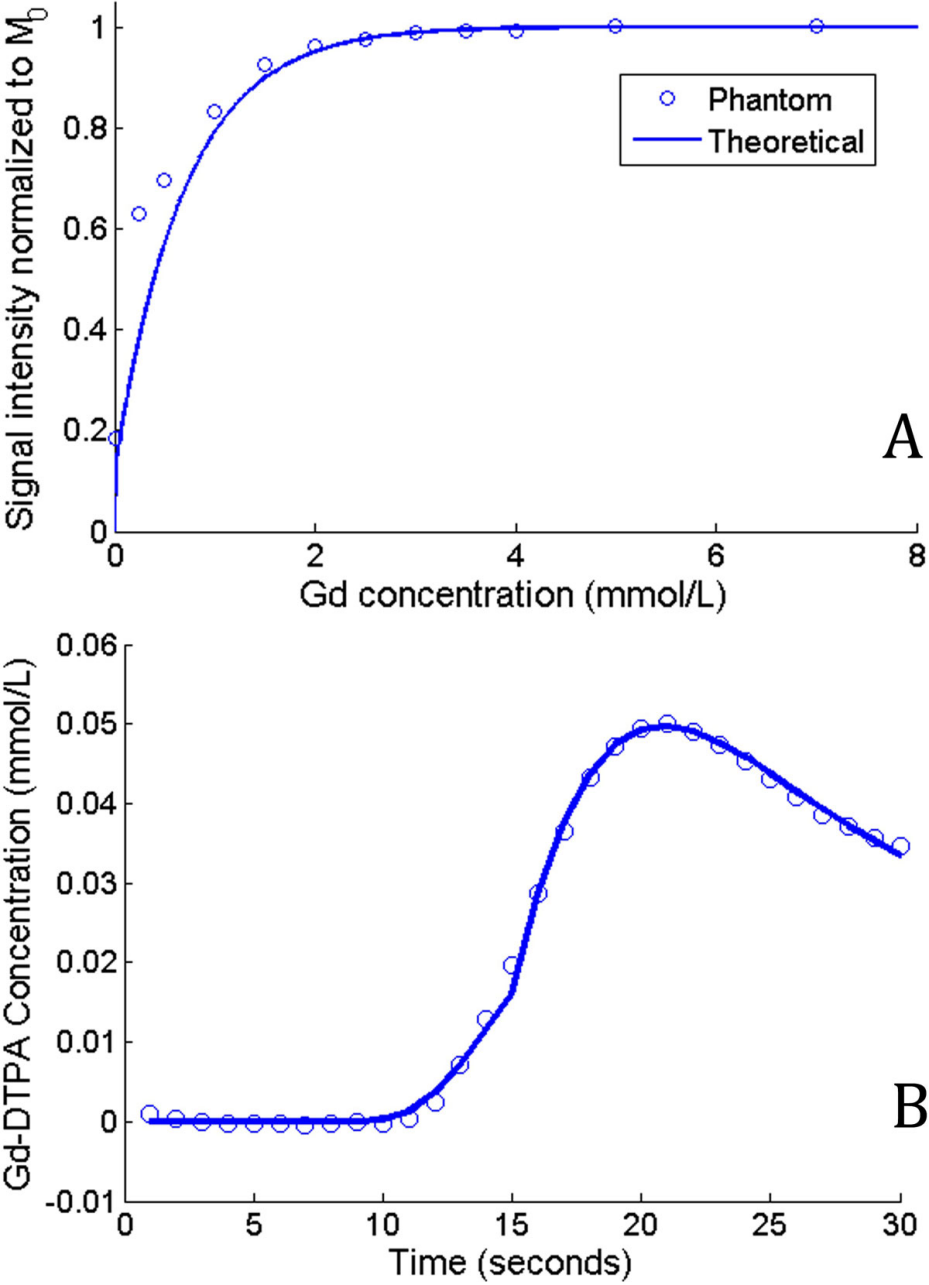
**Gadolinium concentration-Signal intensity curves show the theoretical (line) and measured (circles) phantom results**. A representative tissue function fit is illustrated in panel B.

**Figure 2:**
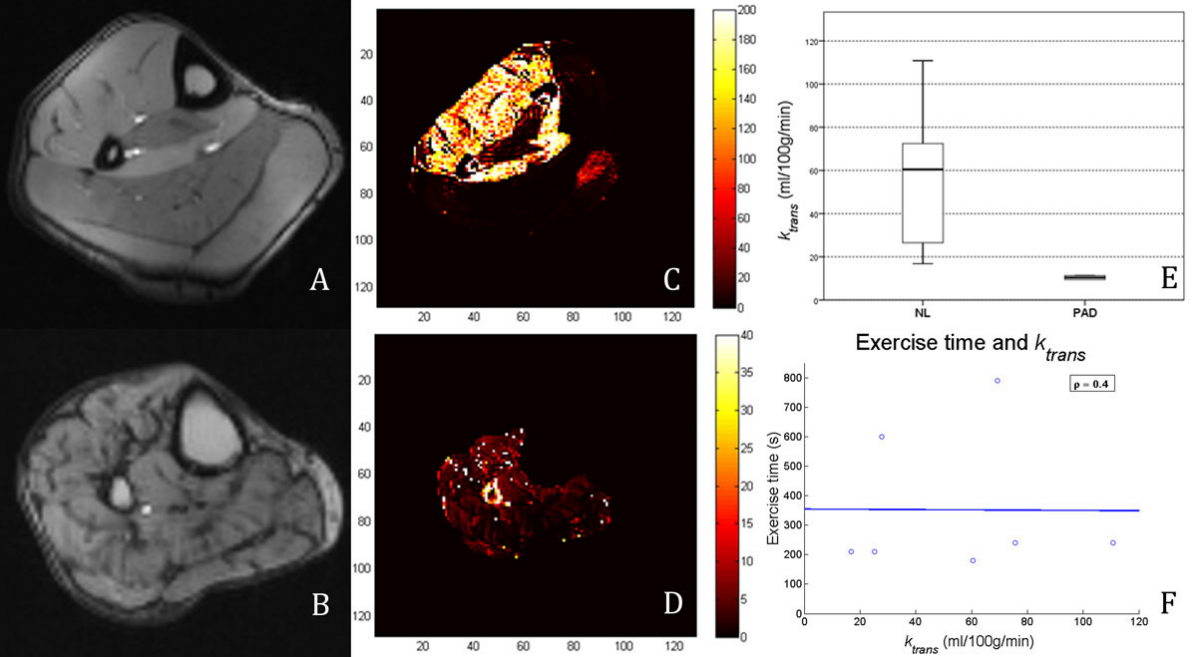
**Representative GRE images at peak intensity of NL (A) and PAD (B) volunteers**. Pixel maps illustrate the differences in *k_trans _*values between NL (C) and PAD (D). Box plot shows the *k_trans _*distribution (E). Note the lack of correlation between exercise time and *k_trans_*. GRE = gradient echo; NL = healthy volunteers; PAD = peripheral arterial disease patients; ρ = Spearman's correlation.

Gd phantom analysis showed good correlation between the theoretical and measured Gd concentrations (Figure 1A). A representative TF curve is shown in Figure 1B. Representative GRE images and calculated pixel maps are illustrated in Figure 2A-2D. Mean *k_trans _*was 55.1 ± 31.3 and 10.4 ± 1.3 ml/100 g/min in NL and PAD respectively (Figure 2E). There was no correlation between exercise duration and *k_trans _*in NL subjects (Figure 2F).

## Conclusions

This pilot study shows that quantitative CE-CMR at peak exercise is feasible. *k_trans _*does not equal blood flow, but instead is a surrogate marker of perfusion modulated by *F *and *E*. Preliminarily, it appears to differentiate normals from PAD as the latter is quite low in PAD. Thus it may become a valuable quantitative parameter for testing novel PAD therapies.

## Funding

T32 EB003841 (DL), NIH HL075792 (CMK).

